# Preliminary RNA-Seq Analysis of Long Non-Coding RNAs Expressed in Human Term Placenta

**DOI:** 10.3390/ijms19071894

**Published:** 2018-06-27

**Authors:** Marta Majewska, Aleksandra Lipka, Lukasz Paukszto, Jan Pawel Jastrzebski, Marek Gowkielewicz, Marcin Jozwik, Mariusz Krzysztof Majewski

**Affiliations:** 1 Department of Human Physiology, School of Medicine, Collegium Medicum, University of Warmia and Mazury in Olsztyn, 10-082 Olsztyn, Poland; mariusz.majewski@uwm.edu.pl; 2 Department of Gynecology and Obstetrics, School of Medicine, Collegium Medicum, University of Warmia and Mazury in Olsztyn, 10-045 Olsztyn, Poland; aleksandra.lipka@uwm.edu.pl (A.L.); marekgowkielewicz@gmail.com (M.G.); prof.jozwik@gmail.com (M.J.); 3 Department of Plant Physiology, Genetics and Biotechnology, Faculty of Biology and Biotechnology, University of Warmia and Mazury in Olsztyn, 10-719 Olsztyn, Poland; pauk24@gmail.com (L.P.); bioinformatyka@gmail.com (J.P.J.)

**Keywords:** placenta, long non-coding RNA (lncRNA), human, pregnancy, high-throughput RNA sequencing (RNA-Seq), transcriptome

## Abstract

Development of particular structures and proper functioning of the placenta are under the influence of sophisticated pathways, controlled by the expression of substantial genes that are additionally regulated by long non-coding RNAs (lncRNAs). To date, the expression profile of lncRNA in human term placenta has not been fully established. This study was conducted to characterize the lncRNA expression profile in human term placenta and to verify whether there are differences in the transcriptomic profile between the sex of the fetus and pregnancy multiplicity. RNA-Seq data were used to profile, quantify, and classify lncRNAs in human term placenta. The applied methodology enabled detection of the expression of 4463 isoforms from 2899 annotated lncRNA loci, plus 990 putative lncRNA transcripts from 607 intergenic regions. Those placentally expressed lncRNAs displayed features such as shorter transcript length, longer exon length, fewer exons, and lower expression levels compared to messenger RNAs (mRNAs). Among all placental transcripts, 175,268 were classified as mRNAs and 15,819 as lncRNAs, and 56,727 variants were discovered within unannotated regions. Five differentially expressed lncRNAs (*HAND2-AS1*, *XIST*, *RP1-97J1.2*, *AC010084.1*, *TTTY15*) were identified by a sex-bias comparison. Splicing events were detected within 37 genes and 4 lncRNA loci. Functional analysis of *cis*-related potential targets for lncRNAs identified 2021 enriched genes. It is presumed that the obtained data will expand the current knowledge of lncRNAs in placenta and human non-coding catalogs, making them more contemporary and specific.

## 1. Introduction

The placenta serves as a metabolic, respiratory, excretory, and endocrine organ, whose proper functioning is required for adequate embryonic development during pregnancy [1]. Fetal growth is a multifactorial and complex process modulated simultaneously by maternal, fetal, placental, and environmental factors predetermined by genetic potential. A properly functioning placenta fine-tunes the expression of various genes essential in pregnancy maintenance and fetal development [2,3]. 

Spatiotemporal expression is a huge impediment in any transcriptome analysis, especially in the placenta, an organ that constantly adapts to feto-maternal environmental alterations. Comprehensive analysis of messenger RNA (mRNA) expression in first- and second-trimester placentas compared to term placentas by microarray assay revealed more genes with increasing than decreasing expression [4]. Furthermore, a source of variability in the placental transcriptome is embryo sex-dependent bias connected with the expression of genes located on the sex chromosomes, which can also affect the expression level of autosomal genes [5]. However, sex-dependent biases in respect to growth, development [6], and predisposition to pregnancy complications are interesting, and placental gene expression regulation remains unclear [5].

Previous studies implied that risk factor profiles for various pathologies are different between singleton and twin births [7]. Furthermore, without any obvious pattern, twin pregnancies are more likely to involve disorders than single pregnancies. Higher perinatal risk, dangerous for both mother and fetus, is associated with the number of embryos in utero. The main risks of multiple pregnancies are early and late miscarriage, preeclampsia (PE), antepartum bleeding, postpartum hemorrhage, preterm delivery, intrauterine growth restriction (IUGR), placental abruption, and stillbirth [8,9,10]. To date, the correlations between gene expression profile and multiplicity of gestation have been studied only in the beaver, and it is suggested that a greater number of fetuses might have a negative influence on pregnancy outcome [2]. 

The great progress of RNA sequencing (RNA-Seq) and the capabilities of numerous bioinformatic approaches make it a powerful technology for thorough transcriptome analysis, which enables characterization of gene expression, alternative splicing events, large-scale discovery of novel transcripts, Single Nucleotide Variant (SNV) prediction, and functional annotation [2,3,11,12]. It is not surprising that in a complex organ like the placenta, there are various distinct transcripts, including mRNA, microRNA (miRNA), and long non-coding RNA (lncRNA), that are not present in other tissues [13]. The lncRNAs are still unexplored ncRNAs characterized by a small number of exons and a sequence length >200 nt that are highly diverse and species-specific with tissue-specific expression [14,15,16,17,18]. LncRNAs act by a range of mechanisms and molecular functions [19], with expression restricted to particular developmental stages [20], and they participate in important biological processes such as embryogenesis [18,21,22], tissue development [23], genomic imprinting [24], and different disease courses [25,26,27]. Given the complex nature of physiological pregnancy, it is important to elucidate possible molecular mechanisms underlying the placental development of male and female fetuses during single and twin pregnancy. 

Disruptions to adaptive changes in the placental transcriptome as a response to altering the feto-maternal environment may be associated with pregnancy complications and compromised fetal outcomes. In this context, defining differences in placenta-specific gene expression regarding the sex of the fetus and the multiplicity of gestation could contribute to the understanding of placental development and function. Since revealing factors that influence the placental expression profile is necessary, this study was conducted to examine whether there are differences in the transcriptomic profile of the human placenta compared for sex of the fetus and number of fetuses. A stringent pathway was applied to identify, analyze, and compare placental transcriptome from male and female fetuses during single and twin pregnancies. This study focused on the lncRNA profile to investigate possible mechanisms regulating the expression profile of the human placenta.

## 2. Results

### 2.1. Characteristics of RNA-Seq Data 

**Figure 1:**
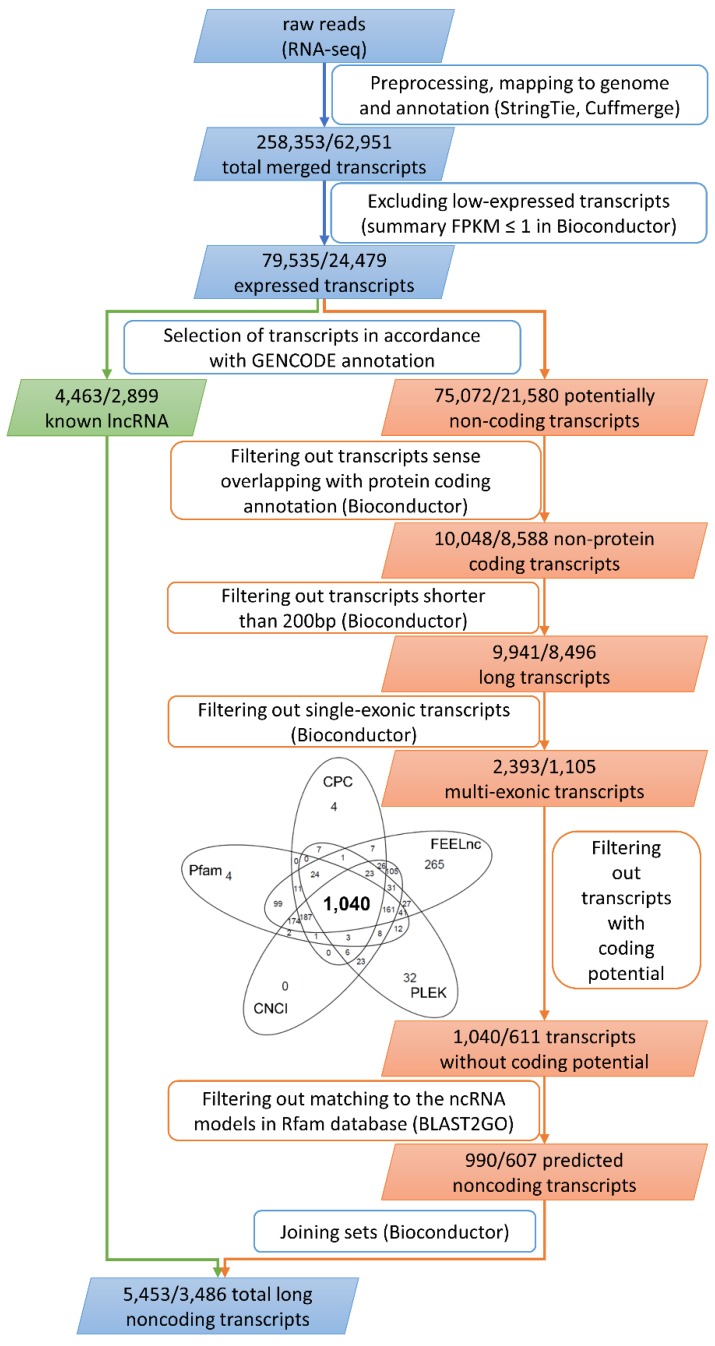
Identification and classification pathway of known (green) and novel (orange) long non-coding RNAs (lncRNAs) expressed in human term placenta; common stages of analysis (blue) join both paths. Numbers in parallelograms refer to amount of lnc transcripts/lncRNA loci. Rectangles show processes and applied tools. Venn diagram presents a number of transcripts without coding potential assigned by Coding-Non-Coding Index (CNCI), Coding Potential Calculator (CPC), FEELnc, Pfam, and PLEK software.

In total, 2 × 119,560,140 raw paired-end reads were generated, and subsequently 2 × 109,363,183 reads were acquired after trimming. The 218,726,366 clean reads were mapped to a reference human genome, and an average of 86.14% reads were mapped uniquely. Among all mapped transcripts (258,353; Figure 1), 67.84% were classified as mRNA, 6.12% were classified as lncRNA, and 2.60% were classified as pseudogenes.

**Figure 2:**
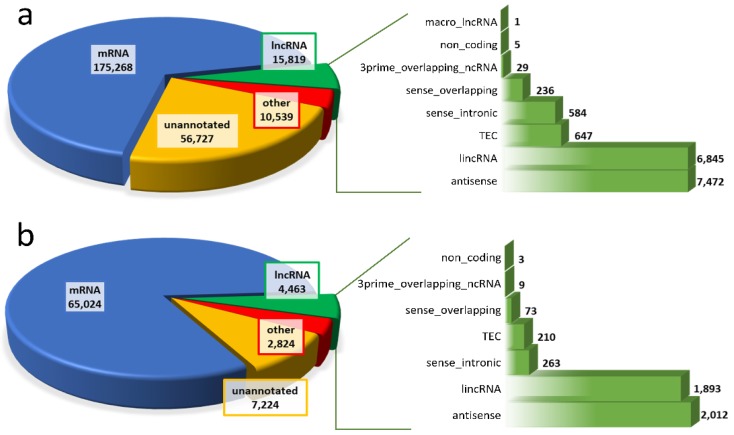
Classification of the assembled human placental transcripts according to their Ensembl code class (pie graphs) detailing lncRNA distribution (bar graphs) of: (**a**) all expressed loci; (**b**) transcripts with expression value (fragments per kilobase of transcript per million mapped reads, FPKM) higher than 1.

Moreover, 1.48% of the expressed transcripts were derived from other RNAs (e.g., processed transcript, Ig genes, or misc RNA) and 21.96% originated from unannotated regions, which included potentially new lncRNA transcripts (Figure 2a). After excluding low expressed transcripts (fragments per kilobase of transcript per million mapped reads (FPKM) ≤1), 79,535 of the identified transcripts (TCONs) were directed for further analysis (Figure 1 and Figure 2b). 

**Figure 3:**
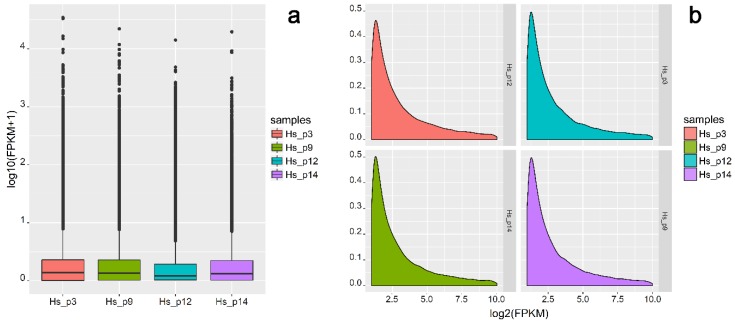
Transcript expression level distribution of each human term placenta sample. (**a**) Box plot of FPKM distribution with different samples on the horizontal axis and logarithmic values of FPKM on the vertical axis; (**b**) density plot of expression distribution with logarithmic values of FPKM on the horizontal axis and density on the vertical axis.

The dynamic range of the expression values was calculated and is presented as a box plot of logarithmic transformed FPKM values for each sample separately (Figure 3a), and the FPKM density distribution is shown in Figure 3b. 

### 2.2. Identification and Profiling of lncRNAs

An lncRNA profile of human term placenta was identified and characterized by applying a stringent pathway (Figure 1). GENCODE enabled selection of 4463 known lncRNAs and 75,072 other than annotated lncRNAs (including 7224 unannotated transcripts; Figure 2b) that qualified for verification of their coding potential and small RNA features (Figure 1). Filtering out sense-overlapping transcripts with protein coding annotation resulted in 10,048 non-protein coding transcripts, corresponding to 8588 potentially non-coding regions. After excluding sequences shorter than 200 nt, 9941 transcripts were obtained. Next, filtering of single-exon variants enabled identification of 2393 multi-exon transcripts. An assessment of coding potential with Coding Potential Calculator (CPC), Coding-Non-Coding Index (CNCI), FEELnc, Pfam, and PLEK generated 1340, 1790, 2222, 1767, and 1439, respectively, for each method (Supplementary Table S1). Intersecting the aforementioned methods allowed determination of the set of 1040 potentially non-coding transcripts (Venn diagram, Figure 1). The remaining transcripts were devoid of non-mRNA sequences, and as a result, 990 variants, corresponding to 607 regions, were classified as predicted lncRNAs. The set of known lncRNAs was composed of 4463 lnc transcripts corresponding to 2899 lncRNA loci. Among them, 2012 were antisense lncRNAs, 1893 lincRNAs, 263 sense intronic transcripts, and 73 sense overlapping (Figure 2b). The classification of the final set of 5453 lncRNA transcripts, according to genomic localization and relation to nearest annotated genes, is shown in Table 1. The 5252 and 201 lncRNA transcripts were distributed within autosomes and sex chromosomes, respectively. Among all 990 predicted lncRNA transcripts, 395 unknown transcripts (Table 1) have not been annotated so far and were deposited (BankIt accession nos. MG828427–MG828821; Supplementary Table S2). 

**Figure 4:**
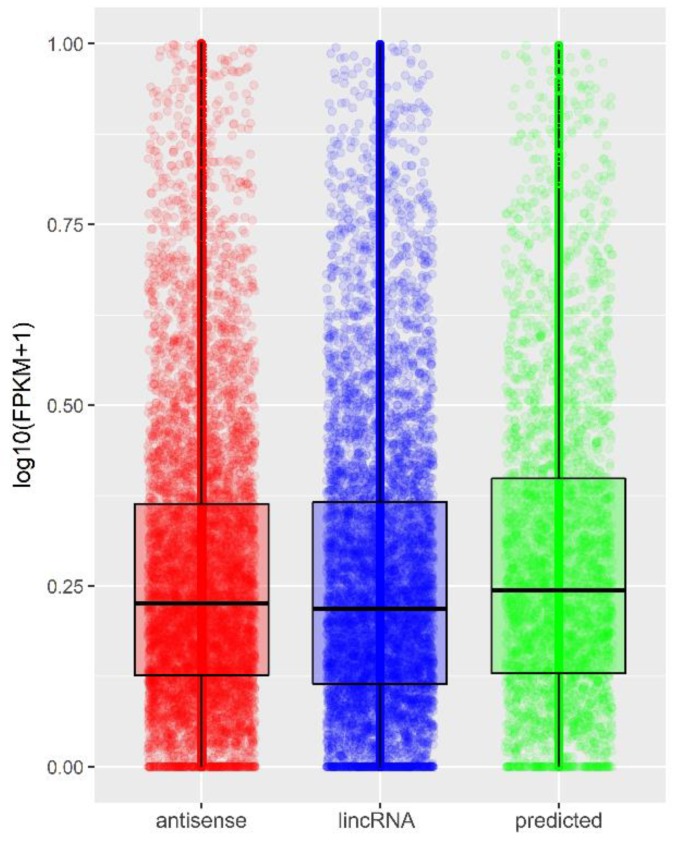
Dispersion of normalized FPKM values presented for the two most numerous lncRNA biotypes: antisense (red), lincRNA (blue), and transcripts predicted as lncRNA (green). Each point represents an individual transcript.

Expression levels of antisense, lincRNA biotype classes, and newly discovered lncRNAs were comparable (Figure 4).

### 2.3. Feature Comparison of lncRNA and mRNA

**Figure 5:**
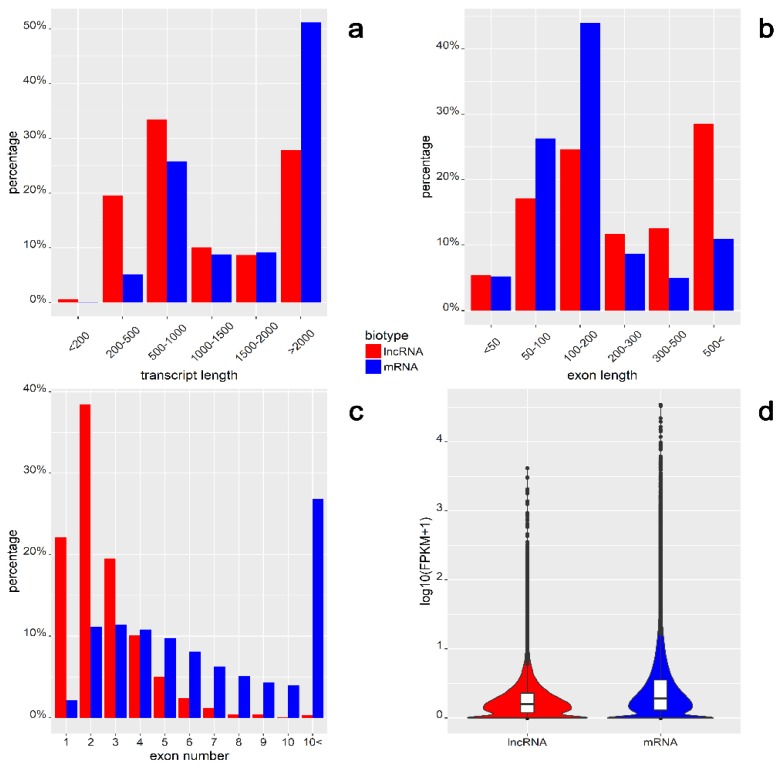
Global summary of comparison between lncRNA (red) and messenger RNA (mRNA) (blue) structural features. lncRNA and mRNA transcripts compared by (**a**) length; (**b**) exon length; (**c**) exon number; (**d**) expression level presented by log10(FPKM + 1); boxes inside each violin plot depict interquartile ranges and individual medians. The differences of average values were statistically significant in each comparison (*p*-value < 2 × 10^−16^ using Welch two-sample *t*-test).

In the current study, 5453 lncRNA and 65,024 mRNA transcripts with FPKM were identified >1. The lncRNA and mRNA transcripts were compared for their total length, exon length, exon number, and expression level (Figure 5). The average length of identified lncRNAs was 1906 nt, while that of mRNAs was 2917 nt (Figure 5a). More than 30% of lncRNAs were in the range of 500–1000 nt, and more than 50% of mRNAs were longer than 2000 nt. Distant length distribution between lncRNA (19.51%) and mRNA (5.18%) was observed in the range 200–500 nt (Figure 5a). The mean exon length of lncRNAs was 737 nt, which was much shorter than mRNAs (337 nt; Figure 5b). Most of the mRNA exons (44%) ranged between 100 and 200 nt, whereas most of the lncRNAs (28.52%) had exon lengths above 500 nt (Figure 5b). The most numerous group of lncRNAs (38.40%) comprised two exons, while only 0.31% of lncRNAs had more than 10 exons, versus mRNAs constituting the largest group (26.86%; Figure 5c). The expression profiles of lncRNA and mRNA biotypes are presented as logarithmic distributions (Figure 5d). The average mRNA expression level was higher than that of the lncRNAs (0.43 vs. 0.31). 

### 2.4. Sex Biases in lncRNA Expression Levels

The expression level (FPKM) of long non-coding transcripts was estimated for both sex and multiplicity biases. A sex-bias comparison revealed five differentially expressed lncRNAs (Table 2; *p*-adjusted < 0.05) and 21 protein-coding genes (Supplementary Table S3). Among the lncRNAs, two loci, XLOC_042918 (chromosome 4) and XLOC_061548 (chromosome X), revealed higher expression levels in female libraries. However, three lncRNA loci, XLOC_050164 (chromosome 6), XLOC_062450, and XLOC_062528 (chromosome Y), were expressed only in male libraries. For protein-coding genes, 11 were upregulated, while 10 were downregulated in female–male comparison (Table S3). The multiplicity-bias comparison did not detect any significant changes in the expression levels of lncRNA and protein-coding genes transcripts. 

### 2.5. Splicing Alterations in Placental Transcriptome

**Figure 6:**
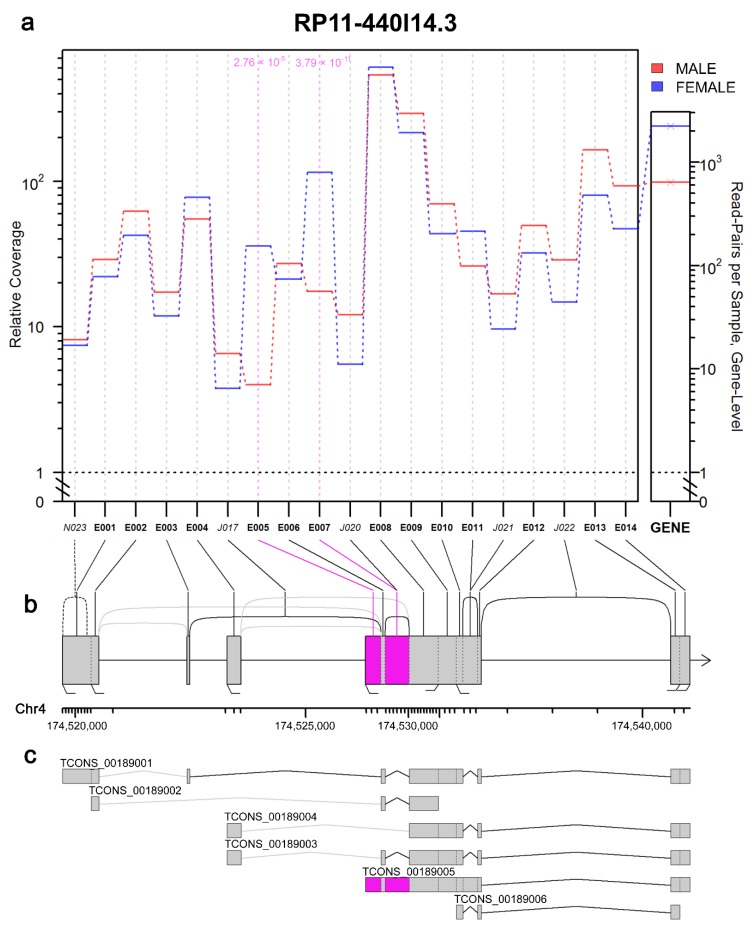
Presentation of differential transcript usage: (**a**) JunctionSeq gene profile plot for *RP11-440I14.3* lncRNA identified in male (red) and female (blue) placental samples. This plot displays estimates for the mean normalized read-pair coverage count for each exon and splice junction. The small panel on the far right displays the total mean normalized read-pair count based on gene level. (**b**) Gene diagram displaying the exonic regions (boxes, labeled E001–E014), known splice junctions (solid lines, labeled J017–J022), and novel splice junction (dashed line, labeled N023) for *RP11-440I14.3* lncRNA localized on chromosome 4 (Chr4). (**c**) The panel shows exon-intron structures of *RP11-440I14.3* variants. Statistically significant differences (*p*-adjust < 0.05) in exon usage are marked in pink.

JunctionSeq allows detection of alternative isoform regulation (AIR) genes, also known as differential transcript usage (DTU). As a result, differentially expressed exons and altered spliced patterns of placental transcripts were detected (male vs. female). Comparing the placental transcriptome from male and female samples revealed 37 AIR/DTU genes displaying 38 and 8 statistically significant differential exon and splice-junction usages, respectively. The use of the JunctionSeq analysis tool led to the detection of new splice junctions in the gene encoding pregnancy-specific β-1-glycoprotein 4 (*PSG4*). Three transcripts with multiple distinct exonic regions, Rho GTPase activating protein 45 (*ARHGAP45*); GATA binding protein 2 (*GATA2*), and long non-coding RNA (*RP11-440I14.3*), were also indicated. Four genes, peptidylprolyl isomerase G (*PPIG*), HLA class II histocompatibility antigen DRB5 beta chain (*HLA-DRB5*), torsin 1A interacting protein 1 (*TOR1AIP1*), and cysteine and serine rich nuclear protein 1 (*CSRNP1*), displayed simultaneous differential exon and splice-junction usage. Among all AIR/DTU events, four significant differential usages of exons were localized within lncRNA loci: *H19*, *AC132217.4*, *RP11-440I14.3*, and *AC005154.6* (Figure 6; Supplementary Table S4). Within *H19*, exon 27 was upregulated in female samples. In female placentas, variable expression of exons 5 and 7 of *RP11-440I14.3* was also observed, although in male placentas, exon 15 of *AC132217.4* and exon 13 of *AC005154.6* were upregulated (Figure 6; Supplementary Table S4).

### 2.6. Functional Analysis of Nearest Neighbor Genes to lncRNAs

**Figure 7:**
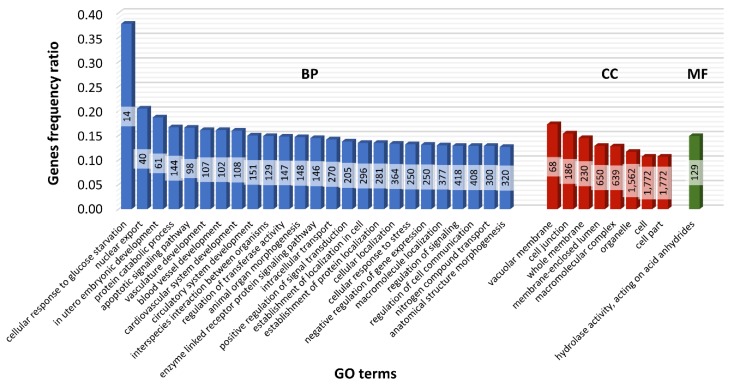
Gene Ontology (GO) annotations (level 1) of *cis* lncRNA target protein-coding genes presenting enriched terms in biological process (BP), cellular component (CC), and molecular function (MF). The height of each bar represents the ratio of target protein-coding genes involved in the particular process relative to all genes associated with a given process in the GO database. The numbers in bars represent the amount of genes involved in a particular GO term.

Potential *cis*-target genes were predicted, revealing possible lncRNA regulation functions in term placental tissues. The genes located within 2000 nt distance (upstream and downstream) from the identified lncRNAs were considered as target genes, and the approach produced 2021 genes. Those genes closely related to lncRNAs were analyzed for Gene Ontology (GO) enrichment, as shown in Figure 7. The majority of *cis*-target genes were enriched (*p* < 0.05) to biological process (148 terms), cellular component (56 terms), and molecular function (20 terms) according to GO classification. GO annotation showed that 61 and 107 protein-coding *cis*-target genes were enriched in in utero embryonic development and vasculature development, respectively. Within the cellular component GO category, 1772 and 1684 *cis*-targets were assigned according to cell and intracellular compartments (Supplementary Table S5). 

### 2.7. Validation of RNA-Seq Results Using External Transcriptomic Datasets

**Figure 8:**
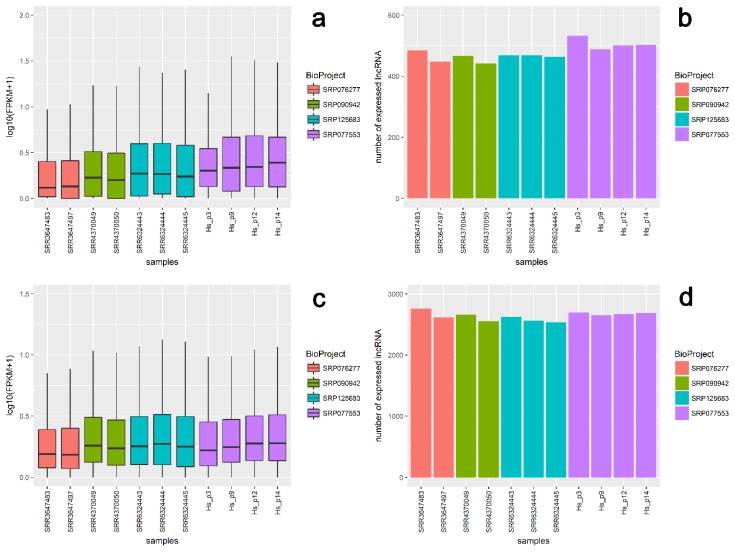
Comparison of transcript expression level distribution between external datasets downloaded from BioProjects SRP076277 (BioSamples SRR3647483 and SRR3647497), SRP090942 (BioSamples SRR4370049 and SRR4370050), SRP125683 (BioSamples SRR6324443, SRR6324444, and SRR6324445), and our dataset (Hs_p3, Hs_p9, Hs_p12, and Hs_p14). (**a**,**c**) Normalized FPKM distribution (box plots) and (**b**,**d**) sum of expression loci (bar graphs) for novel (**upper panel**) and known (**lower panel**) lncRNAs.

Validation with external data confirmed the presence and expression tendencies of the majority of novel (607) and known (2899) lncRNA loci predicted in this study (Figure 8). For external data, mean expression values in logarithmic scale ranged between 0.31 and 0.44 for newly discovered lncRNAs, and between 0.31 and 0.42 for known lncRNA loci (Figure 8, Table 3). Mean expression values for our data ranged from 0.43 to 0.51 for new loci and from 0.37 to 0.42 for known lncRNA regions (Figure 8, Table 3; Supplementary Table S6). Expression levels for 1276 highly expressed lncRNA loci (with FPKM > 2 in at least half the samples) showed that 142 novel and 610 known lncRNA loci had the same high expression profile in external data and our data. As the results obtained for external data were largely consistent with our results, it may further indicate the reliability of the results obtained in this study.

## 3. Discussion

Placenta fine-tunes the expression of various genes involved in major molecular mechanisms essential in pregnancy maintenance and fetal development [2,3,28]. For this reason, any alterations in expression and further processing of specific genes may be correlated with impaired placental function and may directly affect pregnancy outcome [29]. Additionally, the expression mechanisms at both the transcriptional and post-transcriptional level are regulated by numerous lncRNA and lncRNA–RNA interactions [30]. Different expression of genes and their regulatory elements can potentially impact many biological processes and might constitute one of the main regulators of molecular pathways within the placenta [28]. To the best of the authors’ knowledge, the expression profile of lncRNA in the human term placental transcriptome has not yet been studied. Therefore, in this study, lncRNA landscape analysis of human term placenta was performed. 

Among the placental transcripts obtained in our study, 67.84% (175,268) were classified as mRNA, 6.12% (15,819) as lncRNA, and 2.60% (6726) as pseudogenes (Figure 2a). In all, 21.96% of variants (56,727) originated from unannotated regions. According to the current data, 4463 known and 990 previously unknown (predicted) lncRNAs are expressed in human term placental tissue (Figure 1). In comparison, RNA-Seq analysis of first-trimester human placenta transcriptome revealed transcript biotypes in the following classes: 77% protein-coding genes, 9.8% long non-coding genes, and 6.5% pseudogenes [31]. However, the current analysis allowed identification of 21 genes with significantly different expression between males and females, compared to 58 genes discovered by Gonzalez et al. [31]. Further, in placentas of severe preeclampsia cases (~27 weeks of gestation), Gormley et al. [32] classified 15,060 transcripts as mRNA, 823 as lncRNA, and 547 as pseudogenes. Moreover, among the 15,646 dysregulated lncRNAs in early-onset preeclampsia placental tissue, 12,195 were categorized as intergenic, 5182 as antisense, and 1352 as intron sense–overlapping sequences [33]. The present study indicates that among 5453 lncRNA transcripts, the set of 2012 (36.90%) antisense placental transcripts was the largest group, together with 1893 lincRNAs (34.71%) located within the intergenic regions (Figure 2b). Similarly, the class of sense-overlapping sequences (73) was among the smallest groups. The results regarding the lncRNA expression profile in human placenta extend and complement the present transcriptomic databases, which enables genome-wide analysis across tissues and conditions [34]. Moreover, validation with external datasets confirmed the obtained results regarding known and novel lncRNA transcript expression in human term placenta. A general comparison of mRNA and lncRNA features, indicating shorter transcript lengths, longer exon lengths, fewer exons, and lower expression levels for lncRNAs, was consistent with studies in other mammals [17,18,35,36,37,38,39]. Nevertheless, the differences between this study and various transcriptomic experiments result from a strict tissue-specificity pattern of lncRNA expression, restricted spatiotemporal specificity, and differences in adopted pathways.

The expression of mammalian lncRNAs is strictly associated with their regulatory role in a tissue-specific manner. Among various tissues, the testis and ovary were indicated as the most enriched in lncRNAs [40], suggesting their huge regulatory potential within the reproductive system. The expression level analysis in this study revealed five differentially expressed lncRNAs enriched within human term placenta only in sex-bias comparison. It was found that the multiplicity-bias comparison revealed no significant changes in lncRNA expression level. Two lncRNA loci, *HAND2-AS1* and X chromosome inactive–specific transcript (*XIST*), displayed higher expression levels in female libraries and three others, *RP1-97J1.2*, *AC010084.1*, and *TTTY15*, were expressed solely in male libraries. *XIST* as X chromosome–specific was highly enriched in the female libraries. *XIST* is a kind of functional lncRNA uniquely involved in the formation of repressive chromatin and regulation of the X chromosome inactivation process by *cis* action [41,42,43,44]. *XIST*’s expression occurs in a spatiotemporal manner, regulating and influencing female development [45]. *HAND2-AS*, as antisense to *HAND2*, may regulate its expression. *HAND2* is a kind of transcription factor that plays a key role, e.g., in vascularization, development, and differentiation of sympathetic neurons [46,47]. Moreover, *HAND2* fosters a level of fibulin-1, which contributes to progesterone action during implantation [48,49,50]. Usually, the majority of lncRNAs exist as single variants [17], but *HAND2* and *XIST* exhibit more variants: 11 and 9, respectively. Therefore, fetal sex-specific expression of the aforementioned lncRNAs and their variants in the placenta might impact proper placental development and function. That is why further molecular insights into their function must be gained to fully discover their implication in pregnancy outcome. There were 21 protein-coding genes differentially expressed in female and male term placentas. Among them, microsomal glutathione transferase 1 (*MGST1*) was identified to have a confirmed role in oxidative stress protection [51]; relaxin family peptide receptor 1 (*RXFP1*) a receptor for relaxin, a key hormone in mammalian pregnancy [52], and semaphorin 3A (*SEMA3A*) play essential roles in preventing nerve fiber growth in the placenta to protect the fetus from external stress [53].

Previous transcriptomic studies performed on beaver discoid placenta revealed that there are differences in gene expression between twin and triple pregnancies and that the number of fetuses may affect pregnancy outcome [2]. It was found that a multiplicity-bias comparison revealed significant changes of lncRNA expression level in human term placenta. It should be mentioned that such changes may appear in earlier pregnancy stages. Additionally, it cannot be excluded that a similar analysis performed on a greater number of samples would reveal multiplicity as a significant factor affecting the placental transcriptome. The present study should be considered as a pilot screen that may be a good starting point for future functional analysis of more groups of samples. A better understanding of the molecular factors and specific biomarkers during single and twin pregnancies that are predisposed to pathology might be helpful in determining effective prevention strategies. Given the complex nature of physiological pregnancy, such studies are needed to continue to elucidate possible molecular mechanisms underlying placental development during single and twin pregnancies.

Alternative isoform regulation (AIR) can enhance transcriptome diversity and gain another biological function of a single gene by events such as alternative splice sites, alternative transcription start sites, methylation, nucleosome occupancy, internal promoters, nonsense-mediated decay, and/or transcript switching [54]. Alternative splicing events, besides increasing transcriptome complexity, may also disrupt processes or generate pathologies [55]. In the present study, 37 genes and 4 lncRNA loci were identified with AIR/DTU between female and male placental samples. This study enabled detection of a novel splice junction in the gene encoding pregnancy-specific beta-1-glycoprotein 4 (*PSG4*). Pregnancy-specific glycoproteins (*PSGs*) are a specific group of highly expressed trophoblast genes crucial for placentation, acting as regulators of trophoblast cell migration, cytokine secretion, and the establishment of uteroplacental circulation [56]. PSGs are the most abundant proteins in the maternal blood in late pregnancy [57]. A decreased PSG level in maternal serum may be associated with spontaneous abortion, intrauterine growth retardation, or preeclampsia [58,59,60]. Human *PSG* loci (*PSG1*–*PSG11*) are enriched with various types of copy number variations, which may be linked with impaired fertility and pregnancy complications such as preeclampsia [61].

Multiple distinct exonic regions were detected in *ArhGAP45* (also named *HMHA1/HA-1*), which functions as a Rho GTPase [62,63]. Rho GTPases are engaged in the proper functioning of the endothelial barrier [64], embryogenesis [65], neural development [66], cytokinesis, and differentiation [67]. *ArhGAP45* mRNA expression is elevated in preeclamptic placentas and is under the control of oxygen accessibility [68]. GATA binding protein 2 (*GATA2*) regulates stage-specific trophoblastic gene expression of the preimplantation human embryo [69,70,71].

A substantial contribution of lncRNAs in placental formation and function is well known; an evident example is *H19*, a placenta-specific lncRNA highly expressed during mammalian embryonic development [72,73,74]. *H19* is implicated in the regulation of human placenta trophoblast proliferation, placental development [75,76], and fetal growth [77,78]. Moreover, the dynamic profile of *H19* expression may support normal pregnancy, while its impaired regulation might promote preeclampsia, early-onset preeclampsia (EOPE), and IUGR [77,79,80]. *AC132217.4* lncRNA, because it affects 3′UTR and enhances expression level, fosters mRNA stability and upregulates expression of IGF2 circulating growth factor, which acts during pregnancy to promote both fetal and placental growth [81].

Differential usage of exons was also detected in lincRNA *RP11-440I14.3*, localized in *cis* position to hydroxyprostaglandin dehydrogenase (*HPGD*). Hydroxyprostaglandin dehydrogenase inactivates prostaglandins E2 (*PGE2*) and D2 (*PGD2*), which affect several biological processes, such as reproduction, differentiation, and inflammation [82]. In the uterus, PGs play a key role in infection-induced pregnancy loss, in which the concentration of this mediator is increased. As AIR/DTU was detected in genes and lncRNAs, whose functions are related to placental and embryonic development, it should be further investigated to indicate whether the expression profile of specific isoforms can affect the proper or pathological pregnancy course. 

GO analysis was applied to explore the function of the *cis*-target genes. A variety of subclasses of ncRNAs, like piRNA, miRNA, siRNA, and lncRNA, have regulatory roles in gene expression [83,84,85,86,87]. In the present research, enrichment analysis of *cis*-related potential targets for lncRNAs identified 2021 genes. The 61 protein-coding genes were found to be regulated by lncRNA transcripts, and GO enrichment showed that they were enriched in in utero embryonic development (GO:0001701), suggesting that predicted lncRNA functions during pregnancy are linked with developmental, growth, and regulation related processes. Generally, annotation with GO terms displays many of the placentally expressed lncRNA transcripts involved in the regulation of various biological processes also implicated in the gestation course. 

Taken together, since the functions of the majority of lncRNAs have yet to be uncovered, tremendous effort should be made to decipher their implication in the course of gestation, placental development, and reproductive disorders. The present research may be used as a resource for functional studies, which is a huge challenge in determining the influence of lncRNAs on reproductive processes. The authors’ previous study [3] established the placental gene expression landscape of human term placenta during uncomplicated single and twin pregnancies. Therefore, it is hoped that the results of this study will broaden the placenta-specific transcriptome database, which will be useful in a functional field of future research.

## 4. Materials and Methods

### 4.1. Research Material

The lncRNA expression profile of human term placenta was compared between the sex of the fetus (*n* = 2) and pregnancy multiplicity (*n* = 2). All procedures regarding tissue collection, the characteristics of placental samples (*n* = 4), RNA extraction, and RNA-Seq were described previously [3]. Briefly, Hs_p3 (male) and Hs_p14 (female) originated from single pregnancies, whereas Hs_p9 (male) and Hs_12 (female) were from twin pregnancies. To identify lncRNAs expressed in human term placentas, cDNA libraries were constructed and sequenced on the HiSeq 2500 Illumina platform (Illumina, San Diego, CA, USA). The raw data were submitted to the National Center for Biotechnology Information (NCBI) Sequence Read Archive (SRA) under accession No. SRP077553. The experimental protocol was approved by the Bioethics Committee of the Warmia-Mazury Medical Chamber (OIL.164/15/Bioet; 2 April 2015) in Olsztyn, Poland.

### 4.2. Transcriptome Assembly and Identification of Novel Transcripts

The quality of reads was checked using the FastQC tool. Preprocessing using a Trimmomatic tool v. 0.32 [88] included the following: removal of Illumina adaptors and poly(A) stretches, exclusion of low-quality reads (Phred cutoff = 20), and trimming of reads to equal 90 nt in length. Next, paired-end clean reads were aligned to the reference human genome (Homo_sapiens.GRCh38.dna.primary_assembly.fa) with annotation (Homo_sapiens.GRCh38.87.gtf) applying the STAR (v. 2.4, https://github.com/alexdobin/STAR) mapper. As a result, a BAM file alignment of the trimmed reads to the reference genome was obtained for each sample. StringTie v. 1.0.4 (https://ccb.jhu.edu/software/stringtie) [89] and Cuffmerge, as part of the Cufflinks tool v. 2.2.1 (http://cole-trapnell-lab.github.io/cufflinks) [90], were applied to expand gene and transcript annotations based on Ensembl human reference (release 90, August 2017). This approach enabled the identification of unannotated regions and novel splice variants expressed in the placenta. An expanded annotation file (merge.gtf) was used for expression calculation (Cuffquant), normalization (Cuffnorm), and differential analysis (Cuffdiff). All transcript sequences were extracted to a FASTA file using a gffread script (Figure 1).

### 4.3. Classification, Characterization, and Validation of lncRNAs 

Low-expressed transcripts with FPKM values ≤ 1 (expression sum of 4 libraries) were excluded from the set of merged transcripts. Next, all transcripts longer than 200 nt were passed for further analysis (Figure 1). Selected transcripts were divided into 2 main datasets: (1) known lncRNA transcripts (biotypes of GENCODE (https://www.gencodegenes.org/) “lincRNA”, “antisense”, “sense_intronic”, “sense_overlapping”, “bidirectional_promoter_lncRNA”, “non_coding”, “macro_lncRNA”, “TEC” (to be experimentally confirmed), and “3prime_overlapping_ncRNA”); and (2) potentially non-coding RNA (unannotated transcripts and other than lncRNAs). The second dataset, including unknown non-coding sequences, was reduced by removing a transcript assigned to the “protein_coding” Ensembl class code. Next, these transcripts were subjected to multi-exon filtering. Transcript coding potential was assessed by several tools: The Coding Potential Calculator (CPC) (http://cpc.cbi.pku.edu.cn) [91], Pfam (https://pfam.xfam.org) [92], CPAT (http://rna-cpat.sourceforge.net) [93], Coding-Non-Coding Index (CNCI) (https://github.com/www-bioinfo-org/CNCI) [94], and PLEK (https://sourceforge.net/projects/plek/files) [95]. CPC (score < 0) enabled the assessment of ORF occurrence (Figure 1). Transcripts that encoded any conserved protein domains were removed, applying the following parameters: CPC (cutoff < 0), Pfam database (*e*-value 10^−5^; release 27), CPAT (cutoff < 0.43), CNCI (cutoff < 0), and PLEK (cutoff < 0). Further, surviving transcripts were searched in Rfam using Blast2GO software (https://www.blast2go.com) [96], to exclude small ncRNAs (rRNAs, tRNAs, snRNAs, snoRNAs, and miRNAs). Sequences of both known and unknown datasets were denoted as the final set of lncRNAs (Figure 1). Obtained data regarding known and novel lncRNAs were validated by comparison with external data generated in similar studies. SRA resources were searched to find projects focused on RNA-Seq of term placental tissues from normal pregnancies ended by cesarean section. Data from the 3 most accurate BioProjects, SRP076277 (BioSamples SRR3647483 and SRR3647497), SRP090942 (BioSamples SRR4370049 and SRR4370050), and SRP125683 (BioSamples SRR6324443, SRR6324444 and SRR6324445), were chosen for further analysis. Then the raw data were processed with the same approach and parameters that were applied to our data analysis. Downloaded data were aligned to the reference human genome (Homo_sapiens.GRCh38.dna.primary_assembly.fa) with a previously generated merged.gtf annotation file. Then, BAM files were sorted by coordinates and used to calculate FPKM values. Expression values for 607 lncRNA loci predicted as novel and 2899 known lncRNA regions were merged and compared with FPKM values obtained for datasets from the aforementioned BioProjects.

### 4.4. Different Expression and Splicing Analysis

The reads assembled to mRNA and lncRNA sequences were normalized to FPKM values using Cuffnorm. Applying Cuffdiff, the corresponding *p*-values were determined for 2 comparisons: sex and multiplicity bias in placental tissue. Thresholds for significantly different expression were set as follows: *p*-adjusted < 0.05 and log2 fold change (log2FC) ≥ 1.0. A structural comparison between lncRNA and mRNA transcripts was performed by custom R bioconductor scripts. The QoRTs/JunctionSeq pipeline [54] was adopted for differentially expressed exons and splice junction analysis (*p*-adjusted < 0.05).

### 4.5. LncRNA Target cis Gene Prediction

Based on the localization of lncRNA in relation to mRNA, *cis* interactions were predicted, since the *cis* role refers to the influence of lncRNAs on vicinity target genes localized within 2000 nt upstream or downstream of each protein coding gene on the same chromosome. Functional enrichment analysis (*p*-adjusted < 0.05) of the potential *cis* target genes was performed by Kobas 3.0 software (http://www.kobas.cbi.pku.edu.cn) [97] including Gene Ontology (GO) (http://www.geneontology.org).

## Figures and Tables

**Table 1:** Classification of 5453 lncRNA transcripts (class code module in Cuffcompare).

Class-Code	Description	Isoform (TCONS)	Locus (XLOC)
“-”	unknown, intergenic region	395	344
“o”	overlapped with existed gene with a dramatic difference in gene structures	208	170
“x”	overlapped with existed gene in an opposite direction	160	150
“i”	located in introns	2	2
“=”	complete match (of known lncRNA)	3747	2698
“j”	potentially novel isoform (of known lncRNA)	941	579

**Table 2:** Differences in expression level of lncRNAs in sex-bias comparison.

Gene_ID	lncRNAVariant ID	Ensembl Gene ID	HGNC Symbol	Gene Name	Biotype	Locus	Samples		Expression Level [FPKM]		log2fc
							Male	Female	Male	Female	
XLOC_042918	11	ENSG00000237125	*HAND2-AS1*	*HAND2-AS1*	antisense	4:173524692-173591465	Hs_p3, Hs_p9	Hs_p12, Hs_p14	2.416	23.984	3.312
XLOC_050164	1	ENSG00000227012	*LINC02527*	*RP1-97J1.2*	lincRNA	6:111900309-111909386	Hs_p3, Hs_p9	Hs_p12, Hs_p14	2.095	0.000	“-Inf”
XLOC_061548	9	ENSG00000229807	*XIST*	*XIST*	lincRNA	X:73792204-73852753	Hs_p3, Hs_p9	Hs_p12, Hs_p14	1.185	90.740	6.259
XLOC_062450	1	ENSG00000229308	*NA*	*AC010084.1*	lincRNA	Y:4036485-4106081	Hs_p3, Hs_p9	Hs_p12, Hs_p14	1.444	0.000	“-Inf”
XLOC_062528	3	ENSG00000233864	*TTTY15*	*TTTY15*	lincRNA	Y:12662333-12692233	Hs_p3, Hs_p9	Hs_p12, Hs_p14	5.355	0.000	“-Inf”

**Table 3:** Summary statistics of logarithm FPKM values for novel and known lncRNAs in external data and our datasets.

		SRR3647483	SRR3647497	SRR4370049	SRR4370050	SRR6324443	SRR6324444	SRR6324445	Hs_p3	Hs_p9	Hs_p12	Hs_p14
**Novel**	**Min.**	0.0000	0.0000	0.0000	0.0000	0.0000	0.0000	0.0000	0.0000	0.0000	0.0000	0.0000
	**1st Qu.**	0.0200	0.0000	0.0272	0.0000	0.0343	0.0527	0.0288	0.1313	0.0843	0.1331	0.1333
	**Median**	0.1178	0.1345	0.2295	0.2124	0.2744	0.2801	0.2483	0.3100	0.3456	0.3519	0.3937
	**Mean**	0.3073	0.3158	0.3851	0.3723	0.4278	0.4400	0.4262	0.4341	0.4693	0.4878	0.5055
	**3rd Qu.**	0.4167	0.4262	0.5216	0.5149	0.6155	0.6120	0.6010	0.5563	0.6927	0.7024	0.7068
	**Max.**	3.5141	3.5084	3.1126	3.0138	2.8940	2.8243	2.7285	3.5662	3.0406	3.6352	2.8402
**Known**	**Min.**	0.0000	0.0000	0.0000	0.0000	0.0000	0.0000	0.0000	0.0000	0.0000	0.0000	0.0000
	**1st Qu.**	0.0810	0.0732	0.1281	0.1034	0.1093	0.1085	0.0909	0.0972	0.1283	0.1410	0.1412
	**Median**	0.1932	0.1882	0.2657	0.2461	0.2649	0.2865	0.2619	0.2273	0.2574	0.2870	0.2867
	**Mean**	0.3159	0.3127	0.3951	0.3755	0.4016	0.4227	0.4067	0.3698	0.3945	0.4150	0.4228
	**3rd Qu.**	0.4016	0.4150	0.5165	0.4978	0.5357	0.5665	0.5464	0.4826	0.5086	0.5361	0.5451
	**Max.**	3.4224	3.3734	3.4381	3.3992	4.5228	5.1047	5.1712	3.7855	3.5651	3.3986	3.5616

Min., minimum; 1st Qu., first quantile; 3rd Qu., third quantile; Max., maximum of log10(FPKM) expression value.

## Supplementary Materials

Supplementary materials can be found at http://www.mdpi.com/1422-0067/19/7/1894/s1.

## Availability of Supporting Data

The sequencing data from this study have been submitted (http://www.ncbi.nlm.nih.gov/sra) to the NCBI Sequence Read Archive under accession No. SRP077553. In addition, all identified lncRNA sequences of the novel transcripts have been deposited in the GenBank (BankIt accession Nos. MG828427–MG828821).
